# Oligomerization, Conformational Stability and Thermal Unfolding of Harpin, HrpZ_Pss_ and Its Hypersensitive Response-Inducing C-Terminal Fragment, C-214-HrpZ_Pss_

**DOI:** 10.1371/journal.pone.0109871

**Published:** 2014-12-12

**Authors:** Pradip K. Tarafdar, Lakshmi Vasudev Vedantam, Rajeshwer S. Sankhala, Pallinti Purushotham, Appa Rao Podile, Musti J. Swamy

**Affiliations:** 1 School of Chemistry, University of Hyderabad, Hyderabad, India; 2 Department of Plant Sciences, School of Life Sciences, University of Hyderabad, Hyderabad, India; Virginia Tech, United States of America

## Abstract

HrpZ—a harpin from *Pseudomonas syringae*—is a highly thermostable protein that exhibits multifunctional abilities e.g., it elicits hypersensitive response (HR), enhances plant growth, acts as a virulence factor, and forms pores in plant plasma membranes as well as artificial membranes. However, the molecular mechanism of its biological activity and high thermal stability remained poorly understood. HR inducing abilities of non-overlapping short deletion mutants of harpins put further constraints on the ability to establish structure-activity relationships. We characterized HrpZ_Pss_ from *Pseudomonas syringae* pv. *syringae* and its HR inducing C-terminal fragment with 214 amino acids (C-214-HrpZ_Pss_) using calorimetric, spectroscopic and microscopic approaches. Both C-214-HrpZ_Pss_ and HrpZ_Pss_ were found to form oligomers. We propose that leucine-zipper-like motifs may take part in the formation of oligomeric aggregates, and oligomerization could be related to HR elicitation. CD, DSC and fluorescence studies showed that the thermal unfolding of these proteins is complex and involves multiple steps. The comparable conformational stability at 25°C (∼10.0 kcal/mol) of HrpZ_Pss_ and C-214-HrpZ_Pss_ further suggest that their structures are flexible, and the flexibility allows them to adopt proper conformation for multifunctional abilities.

## Introduction

Harpins are a class of proteins produced by certain Gram-negative plant pathogenic bacteria [1], [2]. These proteins are unique and possess multifunctional roles, ranging from virulence factors of bacterial pathogens, plant growth enhancers etc. [3]. In addition, harpins induce a strong defense response in plants, called hypersensitive response (HR), characterized by local programmed cell death [3], [4]. Although harpin was first isolated from *E. amylovora* [1], a variety of other bacterial plant pathogens such as *Pseudomonas syringae*, *Xanthomonas axonopodis*, *E. chrysanthemi*, *E. carotovora*, also produce harpins [5]–[8]. However, the molecular mechanisms involved in elicitation of HR in non-host plants or activation of defense response by the harpins from different phytopathogenic bacteria are largely unknown. Nonetheless, there are several proposals on HR elicitation based on experimental observations [3]. *Pseudomonas syringae* pv. *syringae*, secretes HrpZ_Pss_, a 34.7 kDa protein, which elicits HR in several plants, including tobacco [5]. Lack of crystal structures, limited knowledge of interaction with other cell surface or effector proteins and absence of significant sequence homology with other proteins or among themselves [9] made it difficult to establish structure-activity relationship in HrpZ_Pss_. Experiments on partial deletion mutation studies revealed that several truncated peptides e.g., peptides containing *N*-terminal 103 amino acids, *C*-terminal 214 amino acids of HrpZ_Pss_ (non-overlapping region) can also elicit a strong HR [10], further complicating understanding the molecular mechanism of HR.

In spite of the lack of significant sequence homology in harpins, they share several common properties such as high content of leucine and glycine, relatively high thermal stability, predominantly helical structure and an ability to form pores in cell membranes [5], [11]-[14]. The preponderance of helical regions and high content of leucine prompted us to look for leucine-zipper-like motif in harpins. In a previous study, we proposed the presence of at least two leucine-zipper-like motifs in HrpZ_Pss_ and other harpins [15] can create a variety of different oligomerization states. We, therefore, proposed that the oligomers could be responsible for pore formation or disturbing membrane physiology and result in HR [15]. According to the proposal, harpins may need to oligomerize in order to achieve the pore forming or membrane disturbing ability or HR elicitation. Haapalainen et al. [14], mapped HrpZ_Psp_ and suggested that the region recognized as an HR elicitor is also indispensable for protein oligomerization, in agreement with our earlier proposal [15]. However, the observation that harpin from *Pectobacterium carotovorum* elicits HR in plants but does not form dimers or oligomers [14], questions the oligomer-oriented proposal. Very recently, Anil et al. [16] investigated some deletion mutants of HrpZ_Pss_, and suggested that some of the leucine zipper-like motifs of HrpZ_Pss_ are not essential to induce HR in tobacco. The HR induced by the mutants was faster than that of HR induced by intact HrpZ_Pss_, possibly due to better exposure of the HR inducing region of the protein, for interaction with the plant receptors.

In light of the above, it remains unclear whether oligomerization is an important characteristic for HR elicitation or not. We, therefore, further characterized HrpZ_Pss_ and an HR-inducing C-terminal fragment, C-214-HrpZ_Pss_ using thermodynamic, spectroscopic and microscopic approaches. The results obtained are expected to be useful in understanding the relation between oligomer formation, pore formation and HR elicitation.

Another important characteristic of harpins is their high thermal stability, which has been exploited to purify harpins from a mixture of other proteins [5]. Although HrpZ_Pss_ and other harpins have been extensively characterized biochemically and by mutational analysis, the factors that contribute to their high thermal stability have not been clearly understood. Differential scanning calorimetry (DSC) is a powerful technique for investigating thermal unfolding of proteins and yields three thermodynamic parameters: the melting temperature (T_m_), the change in enthalpy (ΔH_c_) and the heat capacity change (ΔC_p_) between the native and denatured states. These parameters can be used to estimate the free energy of stabilization or free energy of unfolding (ΔG), and subsequently, a protein stability curve can be drawn, which describes the temperature-dependent variation of stability [17]. Since the conformational stability curves of HrpZ_Pss_ and other harpins are not known, our further aims are: 1) to determine the conformational stability of HrpZ_Pss_ and C-214-HrpZ_Pss_, and 2) to understand how the two proteins achieve high unfolding temperature. The results obtained indicate thermal unfolding of these proteins is complex and involves multiple steps and that leucine-zipper-like motifs may take part in their oligomerization.

## Materials and Methods

### Protein expression and purification

HrpZ_Pss_ was expressed and purified as described earlier [15]. The C-terminal *hrpZ* nucleotide sequence (642 bp) encoding the polypeptide with C-terminal 214 residues of HrpZ_Pss_ (C-214-*HrpZ_Pss_*) was cloned under *Nde*I and *Eco*RI sites of pET 28a vector (Novagen). *E.coli* BL21 (DE3) cells transformed with pET 28a- C-terminal-*hrpZ* were grown in Luria Bertani (LB) broth containing kanamycin (50 µg/mL) to OD_600_∼0.5 and induced with 1 mM IPTG. After 3 h of induction, the bacterial cells were pelleted, washed and resuspended in 10 mM sodium phosphate buffer (pH 7.5) and immediately sonicated (1 min pulse on and 30 sec pulse off, 7 cycles, Bandelin MS-72 probe). The sonicate was centrifuged at 14000 rpm for 20 min to remove cell debris and the supernatant was loaded onto a Ni-NTA matrix (Sigma) and washed with 20 mM imidazole. Bound C-214-HrpZ_Pss_ was eluted with 200 mM imidazole in phosphate buffer and then subjected to gel filtration on a Superdex 75 column (GE Life sciences), which was pre-equilibrated with 10 mM sodium phosphate buffer (pH 7.5) in order to free it from imidazole. The eluted protein was concentrated using an Amicon filter (10 kDa cut off, Millipore). Purity of the protein was checked by SDS-PAGE on a 12% gel and by ESI-MS (S1, S2 Figures).

### Circular dichroism spectroscopy

CD spectra were recorded on a Jasco J-810 spectropolarimeter (Jasco International Co., Ltd., Tokyo, Japan, website: http://www.jascoint.co.jp), equipped with a Peltier thermostat supplied by the manufacturer. Samples were prepared in 5 mM sodium phosphate buffer (pH ∼7.5) and placed in a 2-mm path length rectangular quartz cell. Spectra were recorded at a scan rate of 20 nm/min with a response time of 0.5 sec and a slit width of 2 nm. Protein concentration was 2.25 µM for measurements in the far UV region (250–190 nm). Each spectrum was the average of 4 accumulations. In order to study the effect of temperature on the secondary structure of the protein, CD spectra were recorded in the far UV region at different temperatures.

### Differential scanning calorimetry

DSC experiments were performed on a MicroCal VP-DSC differential scanning microcalorimeter (MicroCal Inc., Northampton, MA, USA), equipped with fixed reference and sample cells (0.545 ml each). Sample and reference solutions, prepared in 5 mM sodium phosphate buffer, pH 7.5, were properly degassed with stirring in an evacuated chamber for 5 min at room temperature and then carefully loaded in the calorimeter cells. DSC thermograms were recorded at a scan rate of 60 degrees per hour in the Celsius scale. A background scan collected with buffer in both cells was subtracted from each scan. The temperature dependence of the molar heat capacity of the protein was further analyzed by using the Origin DSC analysis software supplied by the manufacturer.

### Fluorescence spectroscopy

Fluorescence emission spectra were recorded on a Spex Fluoromax-4 fluorescence spectrometer. Slit widths of 2 nm were used on both excitation and emission monochromators. Protein samples (∼0.1 OD at 280 nm) in 5 mM sodium phosphate buffer (pH ∼7.5) were excited at 280 nm and the emission spectra were collected from 290 nm. All the spectra were subjected to buffer-correction before the analysis.

### Atomic force microscopy

AFM measurements were performed as described before [18]. Briefly, a drop of a 0.5-0.75 mg/ml protein solution was placed on a freshly cleaved mica sheet and dried immediately with a gentle stream of nitrogen gas. The salt deposits were removed by extensive washing with milli-Q water. The sample was once again dried with nitrogen gas. All images were recorded in air under ambient conditions in semi-contact mode with a scan rate of 0.8 Hz using a SOLVER PRO-M AFM instrument (NTMDT, Moscow). The force was kept at the lowest possible value by continuously adjusting the set-point and feed-back gain during imaging. Image analysis was performed using NOVA software, supplied by NTMDT.

### Tertiary structure analysis

The amino acid sequence of C-214-HrpZ_Pss_ was submitted to I-TASSER server (http://zhang.bioinformatics.ku.edu/I-TASSER/) in order to build 3-dimensional structural models. The structures were analysed using PYMOL software.

## Results

### Differential scanning calorimetry

**Figure 1:**
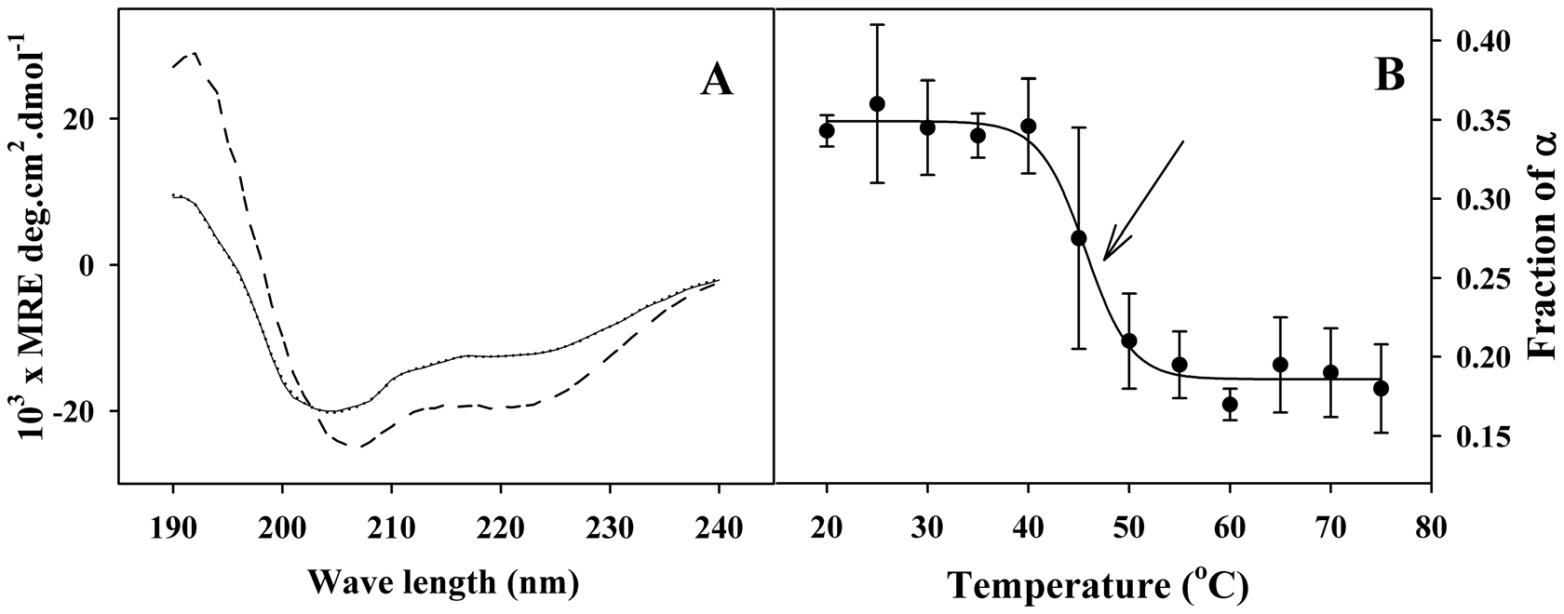
Circular dichroism spectroscopy. A) Far-UV CD spectra of C-214-HrpZ_Pss_ (solid line) and full length HrpZ_Pss_ (dashed line) at 25°C. The dotted line corresponds to the calculated fit obtained by using the CDSSTR program for C-214-HrpZ_Pss_. B) Temperature dependence of α-helical content in C-214-HrpZ_Pss_.

**Figure 2:**
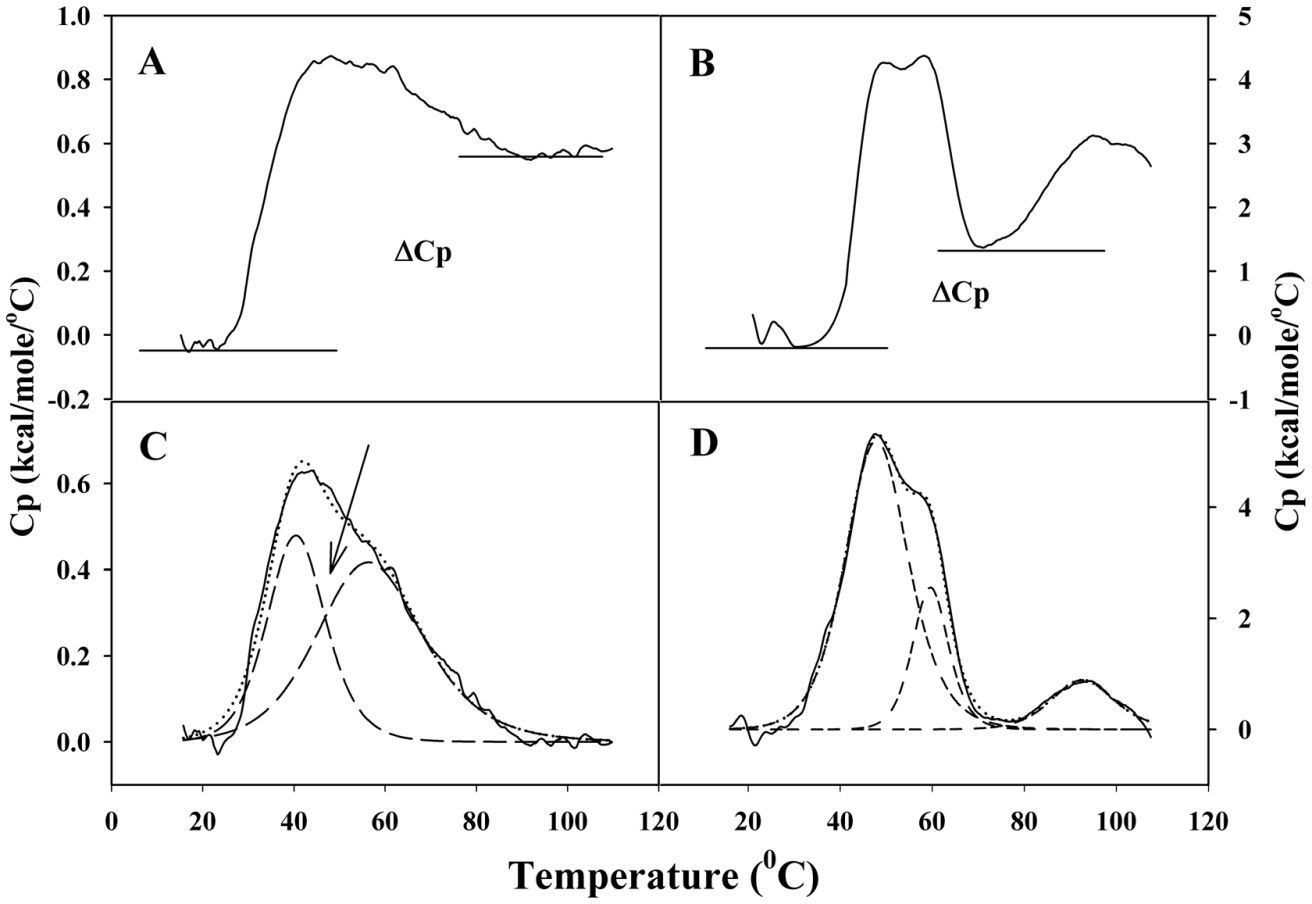
Differential scanning calorimetry. DSC thermograms of C-214-HrpZ_Pss_ (A) and full length HrpZ_Pss_ (B). The scan rate was 60^o^.h^−1^. The change in heat capacity (ΔC_p_) of the native protein and the denatured state are shown. Deconvolution of DSC thermogram of C-214-HrpZ_Pss_ (C) and full length HrpZ_Pss_ (D). The experimentally obtained thermograms are shown as solid lines, individual transitions deduced from deconvolution analysis are shown as dashed lines and the sum of the transitions obtained from deconvolution analysis are shown as dotted lines.

In the present work, we have performed DSC studies on C-214-HrpZ_Pss_ in order to investigate its thermal unfolding mechanism and thermodynamic stability and compare the results with those corresponding to full length HrpZ_Pss_. A heating thermogram of C-214-HrpZ_Pss_ is given in Fig. 1A. For comparison, the thermogram for full length HrpZ_Pss_, taken from our previous study [15] is given in Fig. 1B. The thermogram of C-214-HrpZ_Pss_ contains a broad asymmetric transition, suggesting the presence of two components (Fig. 1A). Thermal unfolding of full-length HrpZ_Pss_ is more complex and contains three distinct transitions centered around 50.0°C, 60.0°C and 94.0°C (Fig. 2B, see [15]). Analysis of the thermogram in Fig. 1A yielded the change in heat capacity (ΔC_p_) of the thermal transition of C-214-HrpZ_Pss_ from the difference between post-transition C_p_ (D) and pre-transition C_p_ (N) baselines of DSC experiments. The average ΔC_p_ obtained from four DSC scans for C-214-HrpZ_Pss_ was 0.57±0.035 kcal/mol/°C. Although there could be some uncertainty in estimating ΔCp from baselines of DSC thermograms, this method has been shown to be consistent with other methods of determining ΔC_p_ [19]. For native HrpZ_Pss_ the unfolding transition occurs through an intermediate and the estimated ΔC_p_ for the transition from the native state to intermediate state was 1.48±0.27 kcal/mol/°C. A stable baseline could not be obtained in thermal scans up to 110°C for the transition from the intermediate to fully unfolded structure (Fig. 1B). Therefore, the ΔC_p_ for this transition couldn't be estimated from the DSC studies; nonetheless, we assume that the structure of the denatured monomer of HrpZ_Pss_ is very close to the structure of the intermediate after transition 2. Indeed, recent fluorescence studies show that the emission λ_max_ of HrpZ_Pss_, observed around 328 nm at room temperature, increases to 347.5 nm at 76°C, suggesting that the tryptophan environment is very close to the aqueous environment at this temperature [20].

Deconvolution of the thermogram of C-214-HrpZ_Pss_ and HrpZ_Pss_ is shown as dotted lines in Fig. 1C. The analysis clearly shows that the broad peak seen in the thermogram of C-214-HrpZ_Pss_ is indeed composed of two different transitions. The 1^st^ transition starts at ∼20°C and ends at ca. 62°C, whereas the 2^nd^ transition starts at about 30°C and ends near 90°C. Values of calorimetric enthalpy (ΔH_c_) and transition temperature (T_m_) for individual transitions, obtained from the deconvolution analysis, are listed in Table 1. These thermodynamic parameters associated with the transitions of HrpZ_Pss_, obtained from our previous study [15], are also listed in Table 1. The total change in calorimetric enthalpy (Δ*H*_tot_) is slightly higher for HrpZ_Pss_ (102.5 kcal/mol) as compared to the C-214-HrpZ_Pss_ (92.1 kcal/mol), suggesting that deletion of N-terminal part (127 aa) of HrpZ_Pss_ results in only a nominal decrease in the calorimetric enthalpy.

**Table 1:** Thermodynamic parameters for the thermal unfolding of C-214-HrpZ_Pss_.

	T_m_ (°C)	ΔH_c_ (kcal.mol^−1^)
Transition 1	39.9±1.1 (50.0)	33.8±6.4 (51.7)
Transition 2	56.9±1.5 (59.9)	58.3±3.0 (36.7)
Transition 3*	(93.6)	(14.1)

Values given are averages obtained from 3 independent measurements. Values in parentheses correspond to the thermal unfolding of full length HrpZ_Pss_ [15]. *C-214-HrpZ_Pss_ does not have transition 3.

### Secondary structure and CD spectroscopy

The secondary structure of C-214-HrpZ_Pss_ was investigated by CD spectroscopy. The far UV CD spectrum of C-214-HrpZ_Pss_ is shown in Fig. 2A (solid line). For comparison, the spectrum of full length HrpZ_Pss_ (dashed line), taken from our earlier study [15] is also shown in this figure. Two minima centred around 204.5 nm and 223 nm were observed for C-214-HrpZ_Pss_, whereas full length HrpZ_Pss_ exhibited two minima centred on 206 nm and 223 nm. These features suggested that similar to full length HrpZ_Pss_, C-214-HrpZ_Pss_ is also a predominantly helical protein. To quantitate the content of different types of secondary structures in C-214-HrpZ_Pss_, the CD spectrum shown in Fig. 2A was analysed by the CDSSTR program using the routines available at DICHROWEB [21]. Reference sets 4 and 7 were used for fitting the experimental spectra and the average values of different types of secondary structures obtained from the analysis are listed in Table 2. The calculated fit obtained using CDSSTR program, shown in Fig. 2A (dotted line), is in excellent agreement with the experimentally obtained spectrum of C-214-HrpZ_Pss_, indicating high accuracy of the analysis. The values obtained at 25°C for the different types of secondary structures are: α-helix (36.0%), β-sheet (14.0%), β-turns (19.5%) and unordered structures (30.0%) (Table 2). For comparison, corresponding data for full length HrpZ_Pss_, taken from our earlier study [15], are also listed in Table 2. Deletion of the 127-residue N-terminal segment from native HrpZ_Pss_ reduces the helical content from 51.5% to 36%. Although the helical content is reduced in the C-214-HrpZ_Pss_ as compared to HrpZ_Pss_, it is still significantly higher than that of other secondary structural elements (Table 2). The secondary structure of C-214-HrpZ_Pss_ is consistent with the proposal that the α-helical structure plays a crucial role in HR elicitation by several harpins and their mutants [6], [15], [22].

**Table 2:** Results of CD spectral analysis of C-214-HrpZ_Pss_.

Program	α-helix (%)	β-sheet (%)	β-Turn (%)	unordered (%)
CDSSTR	36.0 (54.4)	14.0 (9.2)	19.5 (12.2)	30.0 (24.4)

The secondary structural components of the full-length HrpZ_Pss_ [15] are given in parentheses.

### Temperature dependent CD studies

The non-two-state nature of the thermal unfolding process of C-214-HrpZ_Pss_, inferred from the DSC studies, was investigated by CD spectroscopy in order to delineate the underlying structural basis. CD spectra of the protein, recorded at various temperatures, were analysed using the CDSSTR program to monitor temperature-induced changes in the content of secondary structural elements, viz., α-helix and β-sheet. Fig. 1B presents the temperature dependence of the α-helical content of C-214-HrpZ_Pss_. Up to 40°C, there is no obvious change in the α-helical content, suggesting that no change occurs in the secondary structure below this temperature. Since the T_m_ of transition 1 was estimated by DSC to be around 40°C, this indicates that during transition 1 there is no detectable change in the secondary structure of the protein. The decrease in helical content above 40°C suggests thermal unfolding of the protein. It appears from this study that during transition 2 major structural reorganization takes place.

### Thermal unfolding and fluorescence spectroscopy

**Figure 3:**
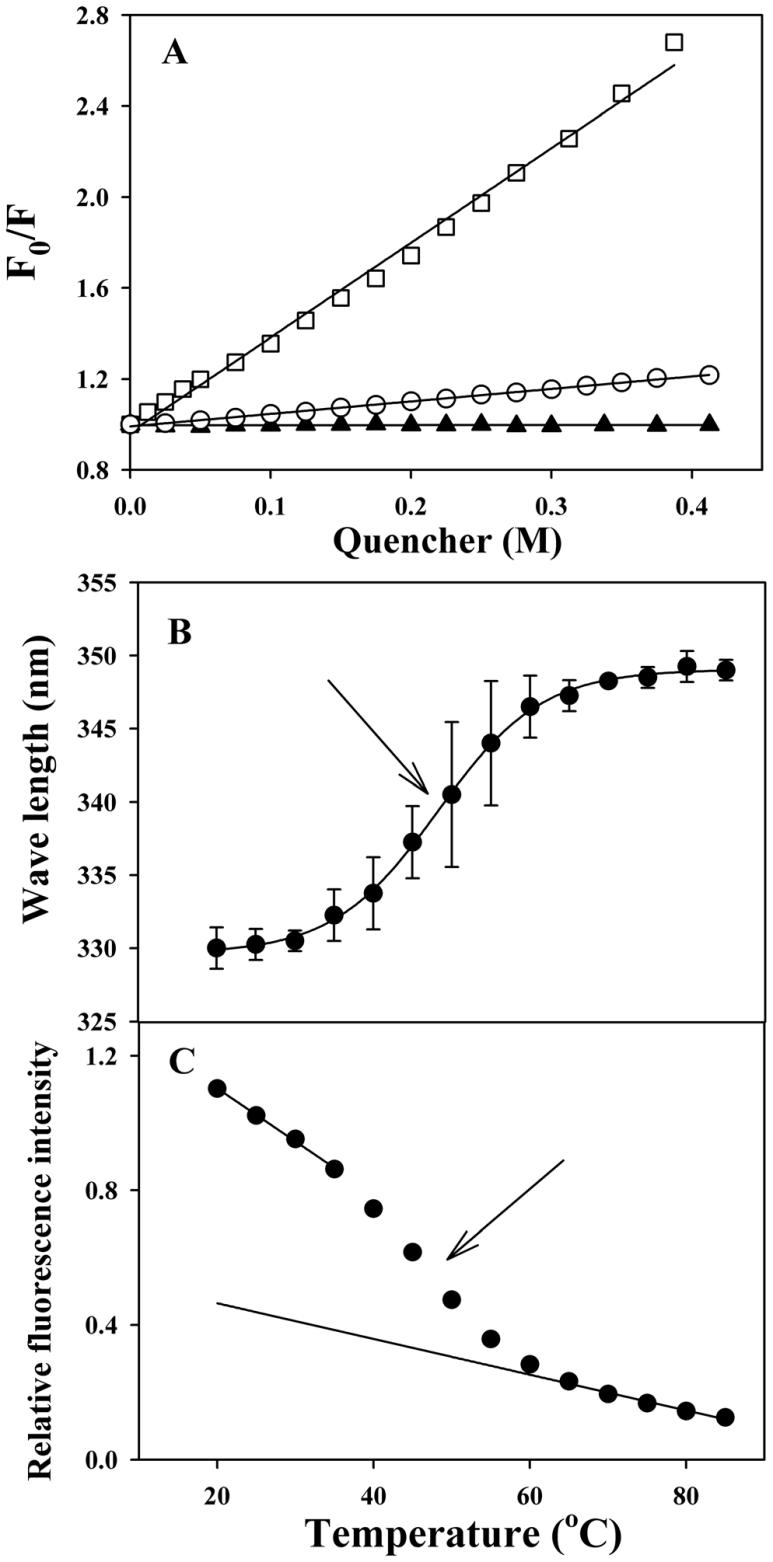
Tryptophan exposure and thermal unfolding. (A) Stern-Volmer plots for the quenching of the intrinsic fluorescence of C-214-HrpZ_Pss_ with acrylamide (□), iodide ion (○) and cesium ion (▴). Temperature dependence of (B) tryptophan emission maximum, and (C) fluorescence intensity at emission maximum, of C-214-HrpZ_Pss_.

The fluorescence spectrum of C-214-HrpZ_Pss_ recorded at 25°C under native conditions shows a maximum at 330 nm, indicating that the Trp residue of C-214-HrpZ_Pss_ is in a hydrophobic environment. HrpZ_Pss_, which contains a single tryptophan has an emission maxima at 327.5 nm [22]. Therefore, the present results suggest that the microenvironment of the tryptophan is more hydrophobic in the native protein. Consistent with this, quenching experiments with neutral acrylamide, anionic I^−^, cationic Cs^+^ and Stern-Volmer analysis to determine quenching constant suggest that tryptophan of C-214-HrpZ_Pss_ is more exposed to the aqueous environment (Fig. 3A, S1 Method, S1 Table). The average lifetime (τ) of single tryptophan in C-214-HrpZ_Pss_ is 2.39ns, which is lower than the reported lifetime (τ) of full length HrpZ_Pss_ (2.79 ns), is also consistent with the tryptophan residue of C-214-HrpZ_Pss_ being slightly more exposed.

Thermal unfolding of C-214-HrpZ_Pss_ was also investigated by monitoring temperature-dependent changes in the emission maximum and fluorescence intensity of the protein (Fig. 3B and 3C). The emission λ_max_ of the native protein does not change significantly up to 35°C, suggesting that only minor changes occur in the microenvironment in this temperature range, whereas above this temperature a steep increase is seen, with the emission maximum reaching a value of ca. 347 nm at 60°C (Fig. 3B). This supports the findings of the CD analysis, which show that up to the midpoint of transition 1 (40°C) no change occurs in secondary structure and that major structural reorganization occurs during transition 2. However, the fluorescence intensity decreases gradually when the temperature is increased and exhibits a sharp decrease near the midpoint of transition 2 (Fig. 3C). At 80°C the emission maximum experiences a further shift to 349 nm, suggesting completion of thermal unfolding, since tryptophan residues that are fully exposed to the aqueous environment, exhibit emission maximum around 350 nm [23]. These observations are in agreement with the results of DSC measurements presented above.

### Aggregation of C-214-HrpZ_Pss_ and atomic force microscopy

Since native HrpZ_Pss_ was found to exist as a soluble polydisperse aggregate in solution [15], [24], it is of interest to investigate the aggregation state of C-214-HrpZ_Pss_. Presence of polydisperse oligomeric structures in protein solutions can be easily detected by native polyacrylamide gel electrophoresis (PAGE). To investigate the aggregation state of C-214-HrpZ_Pss_ in solution and to compare it with native HrpZ_Pss_, we have performed gel electrophoresis of the two proteins and a representative gel picture is shown in S3 Figure. The gel picture shows multiple bands for HrpZ_Pss_ and C-214-HrpZ_Pss_, suggesting that both proteins form polydisperse oligomers, i.e. they exhibit heterogeneity in the oligomerization status. Since C-214-HrpZ_Pss_ was reported to elicit HR in plants [10], this observation is in agreement with our proposal that oligomer formation may play an important role in HR elicitation by harpins [15].

**Figure 4:**
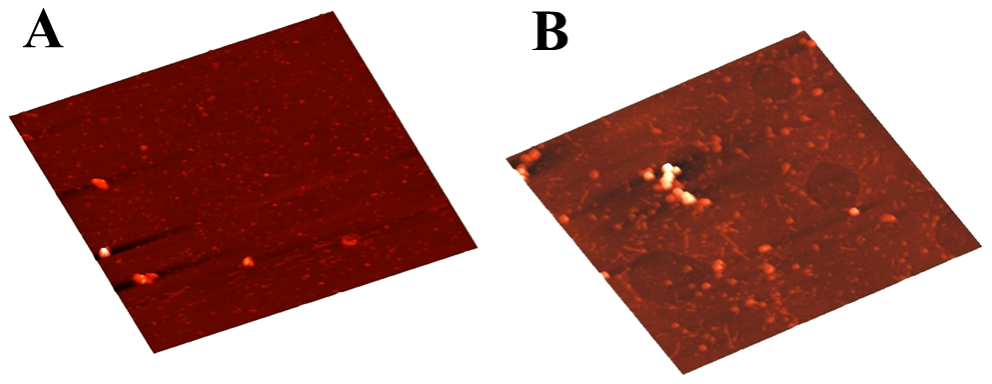
Differential oligomerization of C-214-HrpZ_Pss_ and full length HrpZ_Pss_ studied by AFM. C-214-HrpZ_Pss_ forms non specific aggregates (**A**), whereas full length HrpZ_Pss_ is characterized by the formation of both non-specific and fibrillar aggregates (**B**). Scale bar: 0.5 µm.

Morphological features of aggregates formed by C-214-HrpZ_Pss_ and HrpZ_Pss_ were also investigated by atomic force microscopy (AFM). Fig. 4**A** and **4B** give AFM images of C-214-HrpZ_Pss_ and HrpZ_Pss_, respectively. Both the images show heterogeneity in size and shape among aggregates of this protein, suggesting the presence of polydisperse aggregates. C-214-HrpZ_Pss_ forms non-specific oligomers ranging from 20 to 40 nm with occasional occurrence of larger oligomers (up to ∼100 nm) (Fig. 4**A**), whereas full length HrpZ_Pss_ forms non-specific oligomers of relatively large size (25 to ∼200 nm) with occasional occurrence of even larger aggregates (up to ∼500 nm) (Fig. 4**B**). Full length HrpZ_Pss_ also forms fibrillar aggregates, which may correspond to protofibrils or rod like aggregates (not shown). Together, the AFM and gel electrophoresis experiments suggest that both C-214-HrpZ_Pss_ and HrpZ_Pss_ form polydisperse oligomeric structures.

### 3-D structure prediction, oligomeric form and leucine-zipper

**Figure 5:**
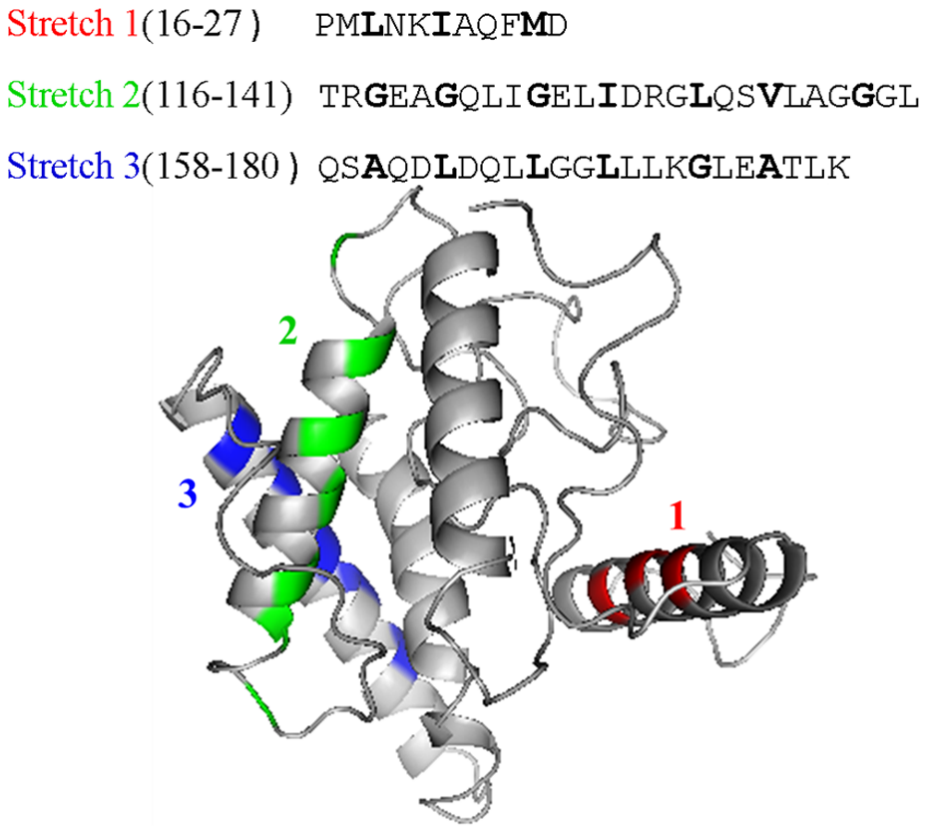
Ribbon representation of the 3D structure of C-214-HrpZ_Pss_. Residues involved in leucine-zipper-like motif or coiled-coil motif are highlighted in red (stretch-1), green (stretch-2) and blue (stretch-3). Amino acid sequences of the leucine-zipper-like motif or coiled-coil motif at various segments of the protein are shown with putative leucine-zipper or coiled-coil forming residues highlighted in bold. Since 127 amino acids were deleted in C-214-HrpZ_Pss_, residue 128 was numbered as 1.

Defining the tertiary structure of harpins has been a challenge due to their polydisperse nature in solution [15]. Therefore, it would be quite interesting to understand the structural basis for the oligomerization/polydispersity of harpins. We proposed earlier that the presence of at least two leucine-zipper-like motifs or coiled-coil motifs in harpins can theoretically nucleate a variety of different oligomeric states. Since AFM and gel-electrophoresis experiments provided strong evidence for the existence of C-214-HrpZ_Pss_ in polydisperse aggregated structure (see above), we expected that at least two leucine-zipper-like motifs to be present in C-214-HrpZ_Pss_. Examination of the primary structure of C-214-HrpZ_Pss_ revealed the presence of three leucine-zipper-like motifs: stretch-1, residues 18-25; stretch-2, residues 118-139; stretch-3, residues 160-177. Sequence alignment, shown in Fig. 5, clearly describes that this particular segment consists of repeats of leucine or other hydrophobic amino acids such as isoleucine, alanine, methionine, and valine at the first (a) and fourth (d) positions. This motif is also a classical coiled-coil motif [25].

In order to know whether the predicted leucine-zipper-like motifs in C-214-HrpZ_Pss_ could form helical structures located at the surface of the protein, such that their interaction between molecules could result in oligomerization, we carried out computational studies to build a 3-dimensional structural model of C-214-HrpZ_Pss_ using the I-TASSER server [26], which was used in our earlier study to predict the possible three dimensional structures of HrpZ_Pss_ and other harpins [15]. A 3-D model of C-214-HrpZ_Pss_, thus obtained shows that the structure of this protein is predominantly made up of helical regions connected by turns and loops (Fig. 5). The amino acid residues involved in the three leucine-zipper-like motifs listed above (residues 18-25, 118-139 and 160-177) are found in helical segments located on the surface of the protein or a small readjustment of conformational angles in the adjacent loop region will render them exposed to the external surface (Fig. 5).

### Temperature dependence of the conformational stability

**Figure 6:**
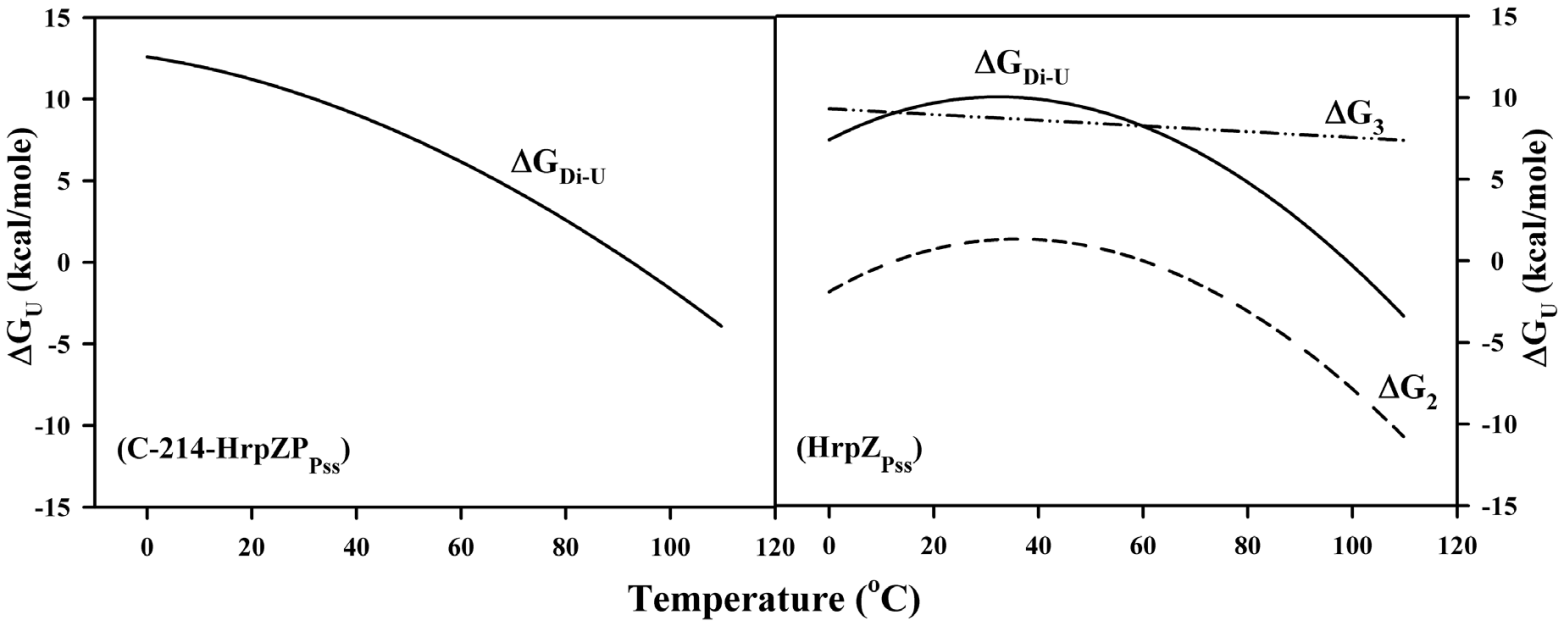
Conformational stability curves of C-214HrpZ_Pss_ and HrpZ_Pss_. Solid lines correspond to *dimer - unfolded state* transition (ΔG_Di-U_). Values of ΔG_2_ and ΔG_3_ for HrpZ_Pss_ corresponding to transition 2 and 3 were calculated using equations 1 and 2 (see text for more details).

The conformation stability curve (i.e. the dependence of ΔG of unfolding with temperature) describes the thermodynamic behaviour of the equilibrium of unfolding. The stability curve of a protein can be drawn with confidence using the modified Gibbs-Helmholtz equation. For N↔U equilibrium, where N is the native state and U is the unfolded state, the equation is: 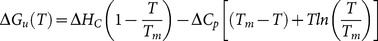
where T_m_ is the melting temperature and ΔH_c_ is the enthalpy of unfolding at this temperature. ΔC_p_ is the heat capacity change, which is independent of solution conditions and temperature between 20 and 80°C [27], [28]. The equation will change for N_2_↔2U equilibrium [29], [30]: 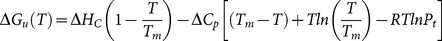
where Pt is protein monomer concentration. Fig 6A and 6B display free energy profiles of C-214-HrpZ_Pss_ and HrpZ_Pss_. The thermal unfolding profile of HrpZ_Pss_ is complex and does not follow a simple two-state transition. Results of studies employing various biophysical approaches suggested the following pathway of unfolding: Oligomer → dimer → partially unfolded dimer → unfolded monomer [15]. Using the Gibbs-Helmholtz equation (1), ΔG for the dimer to partially unfolded dimer (ΔG_2_) at 25°C was estimated to be 1.04 kcal/mol (Fig. 6B). In obtaining the ΔG_2_ we assume that there is no significant change in heat capacity (C_p_) between transition 1 and transition 2. The first transition was attributed to the dissociation of oligomer with different oligomerization states to dimer [15]. HrpZ_Pss_ exists as polydisperse oligomers and the oligomer nucleates from dimer [14]. The secondary structure of the protein at 25°C (before the onset of transition 1) and at 45°C (before the onset of transition 2) is similar and the fluorescence emission maximum of the protein at both the temperatures was identical [22], suggesting that the C_p_ approximation is valid. The ΔG_3_ of HrpZ_Pss_ was determined using equation 2 and plotted as dash-dotted line (Fig. 6B). Since we could not determine C_p_ of unfolded monomer accurately, we keep ΔC_p_ as zero. It is a reasonable approximation as the tryptophan environment of the partially unfolded dimer is very close to that of the unfolded monomer [22]. ΔG_Di-U_ of HrpZ_Pss_ was plotted as solid line by combining ΔG_2_ and ΔG_3_. The ΔG_Di-U_ for C-214-HrpZ_Pss_ was determined using equation 2. For both proteins, the ΔG_Di-U_ at 25°C is comparable; 9.9 kcal/mol for HrpZ_Pss_ and 10.7 kcal/mol for C-214-HrpZ_Pss_.

## Discussion

CD studies show that the secondary structure of HrpZ_Pss_ and C-214-HrpZ_Pss_ is predominantly α-helical, although the α-helical content in C-214-HrpZ_Pss_ (36%) is lower than that in the full length protein (51.5%) (Table 2). The loss in the helical content could be due to the deletion of N-terminal 127 amino acids or because the 3D structure of C-214-HrpZ_Pss_ differs from the HrpZ_Pss_. If the latter proposal is true then one can argue that how different 3-D structures elicit the similar biological activity? It is possible that there is a particular segment of 10–20 amino acids which is responsible for the activity and although the overall structure is not conserved, the structure of the active site is conserved. What are the active sites of HrpZ_Pss_? We have predicted a possible 3-dimensional structure of HrpZ_Pss_ and showed that there are certain helical segments located on the surface of the protein that contain hydrophobic amino acids, which can in principle nucleate the formation of oligomers. Recently, Haapalainen et al. [14] have constructed a series of mutants of HrpZ_Pph_ for functional mapping and found that a 24-amino-acid fragment (290-313) is sufficient to elicit a HR in tobacco. Interestingly, we suggested that amino acid stretch (304-337) of HrpZ_Pph_ may be responsible for oligomerization and biological activity [15]. The results of Haapalainen et al. [14] are in accordance with our proposed model that leucine-zipper-like motifs are responsible for oligomer formation and HR elicitation. If the oligomer-oriented model is correct, then the truncated peptide of HrpZ_Pss_ (C-214-HrpZ_Pss_), which elicits HR, should also form oligomers. AFM and gel-electrophoresis experiments were carried out with HrpZ_Pss_ and C-214-HrpZ_Pss_ to detect oligomerization. The AFM images of HrpZ_Pss_ and C-214-HrpZ_Pss_ suggest the presence of oligomeric structure. The size of individual aggregates is around 20–25 nm for HrpZ_Pss_, which is comparable to the reported average hydrodynamic radius (R_h_) of 20.5±6.2 nm at 25°C for the native protein [15]. The average R_h_ of a monomeric protein with 341 amino acids can be predicted from the following equation [31]:
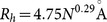



The predicted R_h_ was 2.58 nm and we can approximate that an oligomer with size 20–25 nm may consist of tetramers to decamers depending upon how the monomers are associated. Experimental studies suggest that the aggregated structures of HrpZ_Pss_ can be as large as decamers, or even up to 16mers [14], [24]. Thus the information drawn from our AFM images of HrpZ_Pss_ correlates well with results reported in the literature. The AFM image of C-214-HrpZ_Pss_ also suggests oligomer formation. The average size of the oligomer is ∼20 nm, much higher than the predicted R_h_ from equation 3 for the monomer (2.26 nm). These observations suggest that the aggregated structures are oligomers wherein the number of monomers will be guided by the orientation of monomers in the oligomer. Gel-electrophoresis studies give us a broad smear with multiple underlying bands rather than a single band, further supporting the polydispersity of HrpZ_Pss_ and C-214-HrpZ_Pss_. The question that arises next is, what are the sequence/structural motifs that are responsible for the oligomerization of C-214-HrpZ_Pss_?

The 3-D model of C-214-HrpZ_Pss_ (Fig. 5) consists of several helices and three of them (residues 18-25, 118-139, and 160-177) contain a leucine-zipper-like or coiled-coil motif. The presence of two coiled coil motifs can form a variety of oligomers with different oligomerization states. We proposed that these segments play an important role in the oligomer formation and biological activity. Recently another group showed that the deletion mutants of HrpZ_Pph_ that failed to form pores in lipid membranes, also had defective oligomerization [14]. Thus, it appears that the leucine-zipper-like motifs, which are expected to be involved in oligomerization of HrpZ_Pss_ and C-214-HrpZ_Pss_, are also responsible for pore formation and associated biological activity. However, not all observations are in agreement with this. Harpin from *Pectobacterium carotovorum* does not show any dimer or oligomer formation, but is able to elicit HR [14]. Deletion mutant of HrpZ_Pph_ (lacking amino acid residues 303-335) can elicit HR, but exhibits defective oligomerization [14]. More recently, Anil et al. [16] created a deletion version by removing three leucine-zipper-like motifs from –N and –C termini of HrpZ_Pss_. This mutant corresponding to residues 90-240 of full length HrpZ_Pss_ is capable of inducing HR [16]. However, we can't rule out the possible presence of more leucine zipper-like motifs in this stretch of amino acids, at least from an analysis of its primary structure (see S4 Figure). Indeed, mutational and deletion studies on HrpN of *Erwinia pyrofolie*, HpaG of *Xanthomonas axonopodis* pv. *glycines*, HrpZ_Pph_ of *P. syringae* pv. *phaseolicola* indicate that the specific amino acid regions with 13-24 amino acids are responsible for HR elicitation [14], [22], [32]. Interestingly, alignment of those regions revealed the putative consensus motif (**L**XX**L**LXX**L**X), which contains a high ratio of leucines [14] and the leucines are in a, d and h positions, which in principle can form a coiled-coil structure. Moreover, the observations with harpin from *Pectobacterium carotovorum*, that does not form oligomers, can be explained in terms of the role of lipid membrane. It is possible that lipid membrane plays an important role in oligomer formation as there are evidences suggesting that lipid membranes assist oligomerization of proteins [33]–[35] and these experiments were carried out without membrane. Therefore, it is possible that oligomerization of harpins via putative leucine-zipper motifs play a role in HR elicitation. However, further studies are required to verify this possibility.

The second goal of our present study was to gain insights on the thermal stability of HrpZ_Pss_ and C-214-HrpZ_Pss_. The thermal unfolding profile of C-214-HrpZ_Pss_ was complex and composed of two transitions. Since C-214-HrpZ_Pss_ is a mixture of polydisperse oligomers, we propose that transition 1 is due to dissociation of oligomers. Support for this comes from temperature-dependent CD and fluorescence measurements, which show that major changes in the α-helical content of the protein or the emission maximum of tryptophan residues of C-214-HrpZ_Pss_ occur only above the midpoint (39.9°C) of transition 1. This suggests that during transition 1 major structural reorganization does not occur and hence transition 1 most likely corresponds to the dissociation of oligomeric structure to dimer. We do not have enough evidence that the intermediate is a dimer or not. Considering that the intermediate of thermal unfolding of HrpZ_Pss_ is a dimer and the oligomers seem to be structures made up of dimers [14], [15], it is reasonable to assume that the intermediate for C-214-HrpZ_Pss_ is also dimer. Transition 2 for C-214-HrpZ_Pss_ starts at 30°C and ends at 90°C. In this temperature range the dimer unfolds to yield unfolded monomers: the helical content decreases continuously and red-shift in the tryptophan maxima was observed (Figs. 1, 3). Thus, the thermal unfolding process of C-214-HrpZ_Pss_ can be explained as involving the following pathway: oligomer→dimer →unfolded monomer.

The conformational stability (ΔG_Di-U_) of the C-214-HrpZ_Pss_ and HrpZ_Pss_ at 25°C are ∼10.0 kcal/mol. This suggests that deletion of N-terminal 127 amino acids does not severely affect the stability of the protein, which is also consistent with one of their common features; high thermal stability. Together, apart from oligomerization, we also expect that the structures of HrpZ_Pss_ and C-214-HrpZ_Pss_ are flexible, which allows them to adopt an appropriate conformation in order to elicit HR or to form oligomer.

## Supporting Information

S1 Figure **SDS-PAGE analysis of C-214-HrpZ_Pss_.** Lane 1, molecular weight markers; lane 2, C-214-HrpZ_Pss_. Numbers on the left correspond to molecular weights of the markers (in kDa).
(TIF)

S2 Figure **MALDI-TOF mass spectrum of C-214-HrpZ_Pss_.**
(TIF)

S3 Figure **Native PAGE.** Lane 1: HrpZ_Pss_. Lane 2: C-214-HrpZ_Pss_.
(TIF)

S4 Figure **Primary structure of HrpZMM1, a HrpZ_Pss_ fragment corresponding to residues 90-240 of the full length protein.** Residues in bold correspond to hydrophobic heptadic amino acids, which could form leucine-zipper-like structures.
(TIF)

S1 Table **Results of fluorescence quenching studies with C-214-HrpZ_Pss_.** The extent of quenching achieved by different quenchers and the corresponding quenching constants are given. The final quencher concentration in each case was 0.4 M.
(DOCX)

S1 Method **Analysis of fluorescence data.**
(DOCX)
