# Supplementary figures and images for: Oligomerization, Conformational Stability and Thermal Unfolding of Harpin, HrpZPss and Its Hypersensitive Response-Inducing C-Terminal Fragment, C-214-HrpZPss
Source: PLoS One. 2014 Dec 12;9(12):e109871. doi: 10.1371/journal.pone.0109871 (PMC4264689; doi:10.1371/journal.pone.0109871)

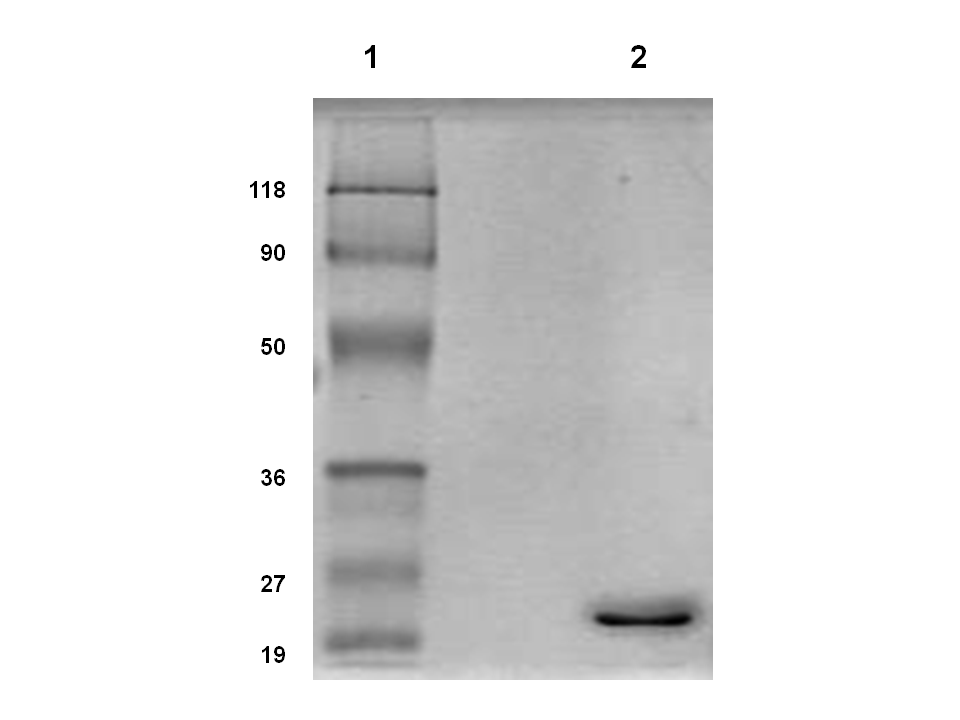

Supplement: S1 Figure — SDS-PAGE analysis of C-214-HrpZPss. Lane 1, molecular weight markers; lane 2, C-214-HrpZPss. Numbers on the left correspond to molecular weights of the markers (in kDa). (TIF) [file pone.0109871.s001.tif]

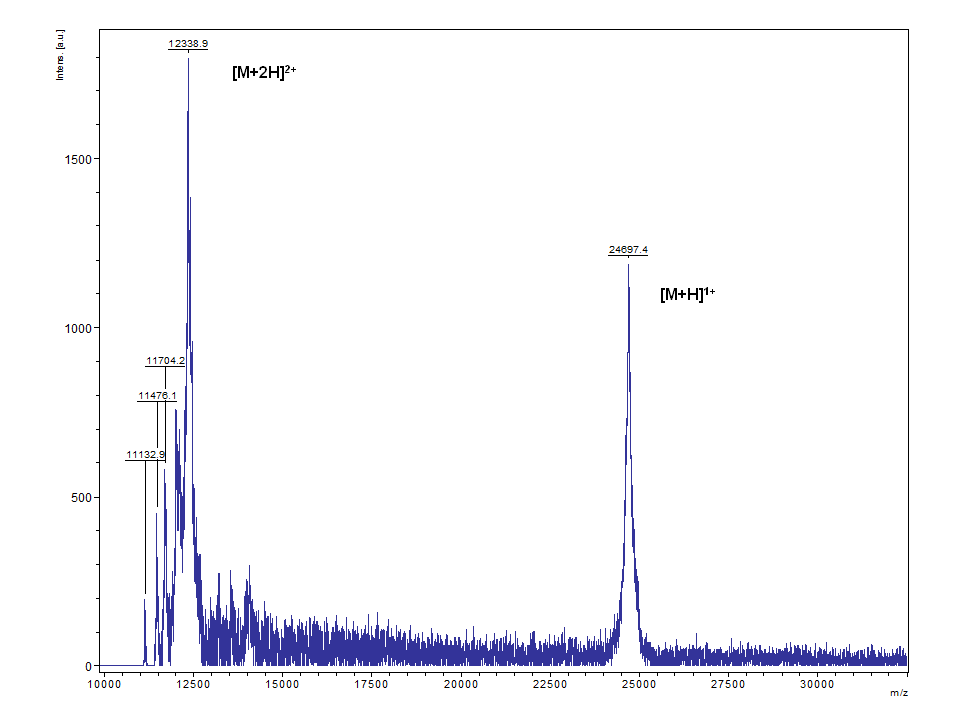

Supplement: S2 Figure — MALDI-TOF mass spectrum of C-214-HrpZPss. (TIF) [file pone.0109871.s002.tif]

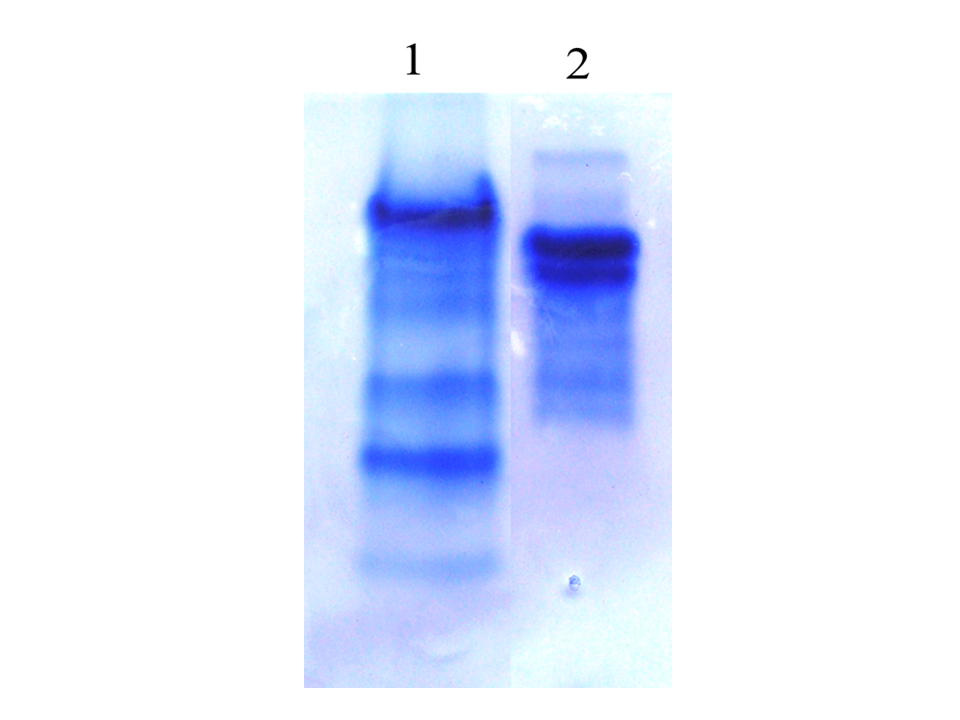

Supplement: S3 Figure — Native PAGE. Lane 1: HrpZPss. Lane 2: C-214-HrpZPss. (TIF) [file pone.0109871.s003.tif]

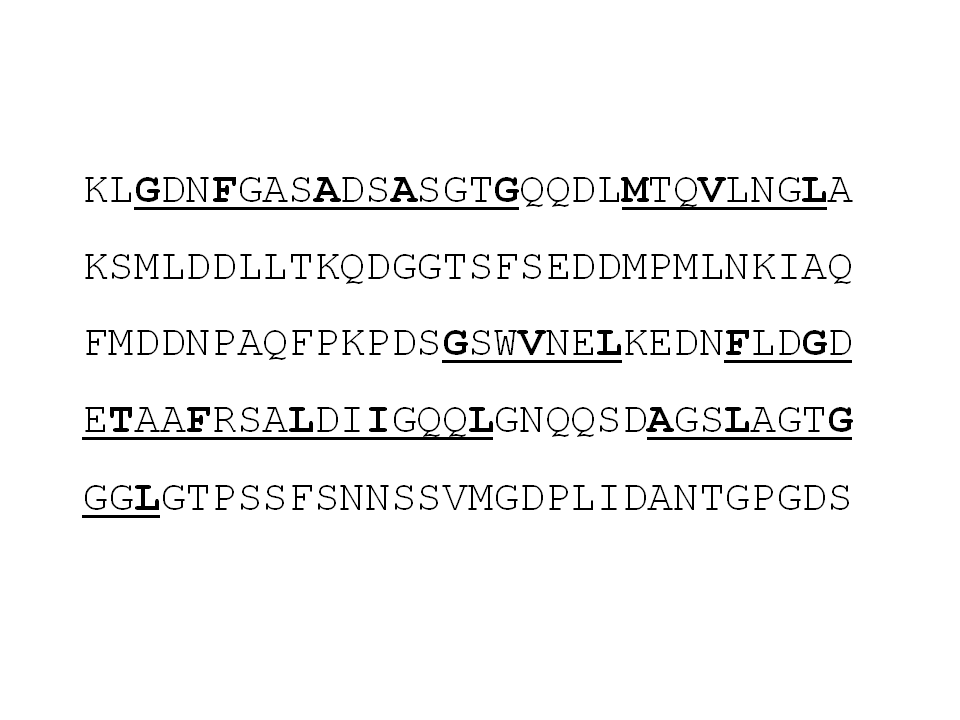

Supplement: S4 Figure — Primary structure of HrpZMM1, a HrpZPss fragment corresponding to residues 90-240 of the full length protein. Residues in bold correspond to hydrophobic heptadic amino acids, which could form leucine-zipper-like structures. (TIF) [file pone.0109871.s004.tif]
