# Supplementary material for: Oligomerization, Conformational Stability and Thermal Unfolding of Harpin, HrpZPss and Its Hypersensitive Response-Inducing C-Terminal Fragment, C-214-HrpZPss
Source: PLoS One. 2014 Dec 12;9(12):e109871. doi: 10.1371/journal.pone.0109871 (PMC4264689; doi:10.1371/journal.pone.0109871)
Supplement: S1 Table — Results of fluorescence quenching studies with C-214-HrpZPss. The extent of quenching achieved by different quenchers and the corresponding quenching constants are given. The final quencher concentration in each case was 0.4 M. (DOCX) [file pone.0109871.s005.docx]

**Table S1**: **Results of fluorescence quenching studies with C-214-HrpZ_Pss_.** The extent of quenching achieved by different quenchers and the corresponding quenching constants are given. The final quencher concentration in each case was 0.4 M.

| **Quencher** | **% quenching** | ***K_SV_* (M^-1^)** |
| --- | --- | --- |
| *Acrylamide* | 63.0 ± 2.0 | 4.4 ± 0.3 |
| *Iodide* | 15.6 ± 2.5 | 0.48 ± 0.1 |
| *Cesium* | 0.0 | 0.0 |
