# Supplementary material for: Oligomerization, Conformational Stability and Thermal Unfolding of Harpin, HrpZPss and Its Hypersensitive Response-Inducing C-Terminal Fragment, C-214-HrpZPss
Source: PLoS One. 2014 Dec 12;9(12):e109871. doi: 10.1371/journal.pone.0109871 (PMC4264689; doi:10.1371/journal.pone.0109871)
Supplement: S1 Method — Analysis of fluorescence data. (DOCX) [file pone.0109871.s006.docx]

**Method S1. Analysis of fluorescence data**

***Stern-Volmer analysis of fluorescence quenching***

The fluorescence data obtained with different quenchers were analyzed by the Stern-Volmer equation:

F_0_/F = 1 + *K_SV_*[Q] (S1)

where F_0_ and F are the representative fluorescence intensities, corrected for dilution, in the absence and presence of quencher; [Q] is the quencher concentration, and *K_SV_* is the Stern-Volmer quenching constant of the protein for a given quencher.

***Fluorescence lifetime measurements***

Fluorescence lifetime measurements were performed on an IBH-5000 single photon counting spectrofluorimeter equipped with a NanoLED excitation source and a cooled microchannel plate photomultiplier tube from Hamamatsu (Model R0839U-50). The time resolution of the spectrometer was ~50 ps. Samples of C-214-HrpZ_Pss_ (OD_280nm_ < 0.1) were excited at 281 nm and emission was monitored at 331 nm. All experiments were performed using excitation and emission slits with a nominal bandpass of 12 nm or less. Lamp profiles were measured at the excitation wavelength using Ludox (colloidal silica) as the scatterer. The signal/noise ratio was optimized by collecting at least 5000 photon counts in the peak channel.

The fluorescence decay curves thus obtained were analyzed by a multiexponential iterative fitting program supplied by IBH to obtain the fluorescence lifetimes (τ_i_) and the corresponding preexponential weighing factors (α_i_). The average lifetime of fluorescence decay for C-214-HrpZ_Pss_ was calculated from these values using the following expression:

τ = Σ_i_α_i_τ_i_ (S2)

where τ is the amplitude average fluorescence lifetime.
